# Disentangling autoencoders and spherical harmonics for efficient shape classification in crystal growth simulations

**DOI:** 10.1038/s42005-025-02129-7

**Published:** 2025-07-02

**Authors:** Jaehoon Cha, Steven Tendyra, Alvin J. Walisinghe, Adam R. Hill, Susmita Basak, Peter R. Spackman, Michael W. Anderson, Jeyan Thiyagalingam

**Affiliations:** 1 Scientific Computing, Rutherford Appleton Laboratory, Science and Technology Facilities Council, Harwell Science and Innovation Campus, Didcot, United Kingdom; 2 CrystalGrower Ltd., Core Technical Facility, Manchester, UK; 3 Department of Chemistry, The University of Manchester, Manchester, UK; 4 School of Molecular and Life Sciences, Curtin University, Perth, WA Australia

**Keywords:** Coarse-grained models, Computational methods, Chemical physics

## Abstract

Controlling crystal growth is a challenge across numerous industries, as the functional properties of crystalline materials are determined during formation and often depend on particle shape. Current approaches rely on expensive, time-consuming experimental studies complemented by exhaustive parameter space simulations, creating significant computational and analytical burdens. Despite machine learning advances in crystal growth for structure-property relationships, applications targeting morphological control remain underdeveloped. Here, we demonstrate how disentangling autoencoders combined with particle aspect ratio and spherical harmonics descriptors can enhance simulation workflows for crystal growth. This approach reveals continuous transformation pathways between different crystal morphologies whilst preserving underlying crystallographic principles. Our method significantly reduces data analytics burdens, shortens design study timelines, and deepens understanding of crystal shape control. This framework enables more efficient exploration of possible crystal morphologies, facilitating the targeted design of crystalline materials with specific functional properties.

## Abstract (web summary)

Crystal growth simulations generate vast datasets that are difficult to analyse using traditional methods alone. By combining disentangling autoencoders with spherical harmonic descriptors, the authors create an interpretable system that maps continuous transformations between crystal morphologies while preserving underlying crystallographic principles.

## Introduction

The crystalline state is central to a significant proportion of the functional materials utilised in our daily lives, whether in pharmaceutical formulations for medicines such as aspirin or paracetamol, the semiconducting silicon found inside our computers, or ‘green’ materials used for hydrogen storage or carbon dioxide removal. Crystalline materials are produced, or grown, in the lab or at an industrial scale through processes that exploit state change. Individual growth units, typically ions or molecules, are added in a block-by-block fashion from a solution, melt, or the gas phase. The precise design and control of crystallisation processes concern a range of factors operating at different length scales. The shape (or morphology) of a crystal is a key mesoscale property established at formation that can affect its overall functionality. In the case of molecular crystals comprising active pharmaceutical ingredients, crystal morphology directly influences properties such as dissolution rates and bioavailability, which in turn impact a drug’s effectiveness^1–4^.

Typically, crystal growth studies comprise expensive and time-consuming high-throughput experimental screening. However, physics-based modelling is increasingly used at different scales, such as ab initio methods to model materials at the quantum level, molecular dynamics to map the evolution of thermodynamic processes involved in crystal growth, or (kinetic) Monte Carlo modelling to simulate the growth of surfaces or whole crystals at atomic resolution. An example of the latter is the CrystalGrower package^5–9^. Using only a limited set of input parameters, a coarse-grained representation of a crystal’s morphology, surface topography, and layer-by-layer growth can be produced on a desktop computer. This can be correlated with experimental results to provide insight into formulation problems and point towards appropriate experimental parameter changes. However, to fully explore even a limited parameter space, tens of thousands of simulations are typically required. This results in large volumes of complex image-based and numerical point cloud data, which are manually analysed. This data burden can prove time-consuming, as useful or relevant data can lie hidden within the parameter space.

Within the last decade, machine learning (ML) methods have made a significant impact in the field of molecular and materials science^10,11^. Specifically, in the field of crystal growth, one of the earliest examples used artificial neural networks and discriminant factorial analysis to classify crystal shapes, finding both methods equally effective in utilising Fourier descriptors and morphological parameters for automated shape recognition, but using a small dataset of 158 real particles^12^. Modern, data-driven approaches utilising structural databases or molecular descriptors have primarily explored structure-property relationships to aid the prediction of morphologies of pharmaceutical crystals^13^, the probability of co-crystal formation^14^, the estimation of crystalline density from chemical structure^15^, and the development of a particle informatics workflow^16^. ML has also served as an emergent tool in crystal structure prediction, having been shown to distinguish chemical elements based on coordination topology in large crystallographic datasets, predict crystal structures with high accuracy across diverse compounds while reducing computational costs, enhance quantum mechanical calculations through data-efficient approaches, replace expensive density functional theory calculations by actively learning on-the-fly, and accurately predict thermal behaviour through anisotropic displacement parameters using graph neural network architectures^17–21^. It has also seen application in solving the crystallographic phase problem, reducing the volume of data needed to resolve the structure of weakly scattering crystals^22^. Large language models (LLMs) have been successfully utilised to generate plausible crystal structures for inorganic materials by training on CIFs, achieving competitive performance with existing methods while offering unique advantages in flexibility and space group-constrained generation^23^. Methods that lean towards the process side of crystal engineering have been developed, including investigating Al-Cu alloy nucleation in situ by combining synchrotron X-ray radiography and ML^24^, predicting interstitial oxygen concentration in Czochralski Si growth^25^, and determining the optimal synthetic conditions as part of a high-throughput perovskite discovery workflow^26^. Machine learning force fields (MLFFs) and interatomic potentials (MLIPs) have also seen application in studying far from equilibrium liquid-to-crystal Si growth and crystal defects^27^. Furthermore, in combining simulation and atomistic modelling with ML, effects such as structural ordering at the solid-liquid interface and atomic dynamics at grain boundaries have been investigated^28,29^.

Other studies have explored the use of ML in applications concerning particle shape outside of the crystal growth domain. Convolutional neural networks have been utilised in the prediction of packing density and flowability of non-spherical particles via the analysis of shape features^30^, whilst image processing and neural network methodology have been developed to characterise the size distribution of gravel particles with a focus on particle boundary delineation and shape feature extraction^31^. In addition, variational autoencoder (VAE) methodology has been used to extract morphological features from primate mandible imaging data for automatic classification and reconstruction^32^. Whilst it is now generally accepted that ML is able to facilitate both the prediction of crystal structures and key physicochemical properties, there remains space for its use in predicting and understanding the range of morphologies available experimentally^33^.

Spherical harmonic shape descriptors (SHSDs) have provided an alternative, effective means of describing shapes, albeit one that can be considered more complex. SHSDs have been used in computer vision for 3D shape recognition^34,35^ and have proved extremely useful in shape-matching applications^36^. The use of SHSDs, mainly as rotationally invariant formalisations, is observed in chemical and biological domains, an example being where the shapes of molecules, proteins, or cells have been successfully described^37–39^. Within evolutionary biology, spherical harmonics have been used to model the shapes of complex morphological structures from continuous surface maps produced by imaging techniques, including computer tomography and confocal microscopy^40^. By contrast, only a handful of studies utilise SHSDs as particle shape descriptions. A spheroidal harmonics approach has recently been demonstrated as an improvement over traditional spherical harmonics for shape analysis of oblate and prolate particles^41^. SHSDs have also been used to characterise and simplify 3D particle morphologies to quantify form, roundness, and roughness for more accurate simulation of granular materials^42^. In combining spherical harmonic analysis with X-ray micro-computed tomography, multiscale morphological features of sand particles were successfully characterised^43^. By using a range of frequency spaces (degrees 2–15), it was possible to effectively represent shape, local angularity, and surface roughness. Finally, the use of spherical harmonics in describing 3D (polygonal) single-crystal shapes has recently been introduced for the comparison of experimental data with simulation, the methodology of which is utilised as part of this study^7^.

In this study, we combine a disentangling autoencoder (DAE) approach with aspect ratio analysis and SHSDs to tackle the challenge of classifying crystal shapes from large simulation datasets. We demonstrate that this integration creates a framework for analysing crystal morphologies in a lower-dimensional latent space, preserving critical shape information while enabling an intuitive visualisation of shape transitions. Our results show that the latent space effectively encodes physically meaningful aspects of crystal geometry that align with crystallographic principles, capturing key relationships between simulation parameters and resulting morphologies. This approach significantly reduces the analytical burden of processing large simulation datasets, provides continuous trajectories between different shape classes, and reveals crystallographic constraints on morphology that would be difficult to observe through traditional analysis methods. The framework not only improves upon existing crystal growth simulation workflows but also establishes a foundation for more efficient crystal shape design and control.

## Results

### Crystal morphologies and particle shape descriptors

A crystalline solid is one that forms a three-dimensional array of its atoms, ions, or molecules with average long-range order. This is a function of the material’s innate molecular-level symmetry. For a given crystal structure, the allowed facets, which are analogous to the faces of a given geometric shape, depend on this symmetry. In addition, a system’s unique chemical properties and external environmental factors, such as temperature or the mother liquor composition, can affect the morphology expressed by a particular crystal structure, whether the presence of certain faceting or the relative proportions of different facet families.

**Fig. 1:**
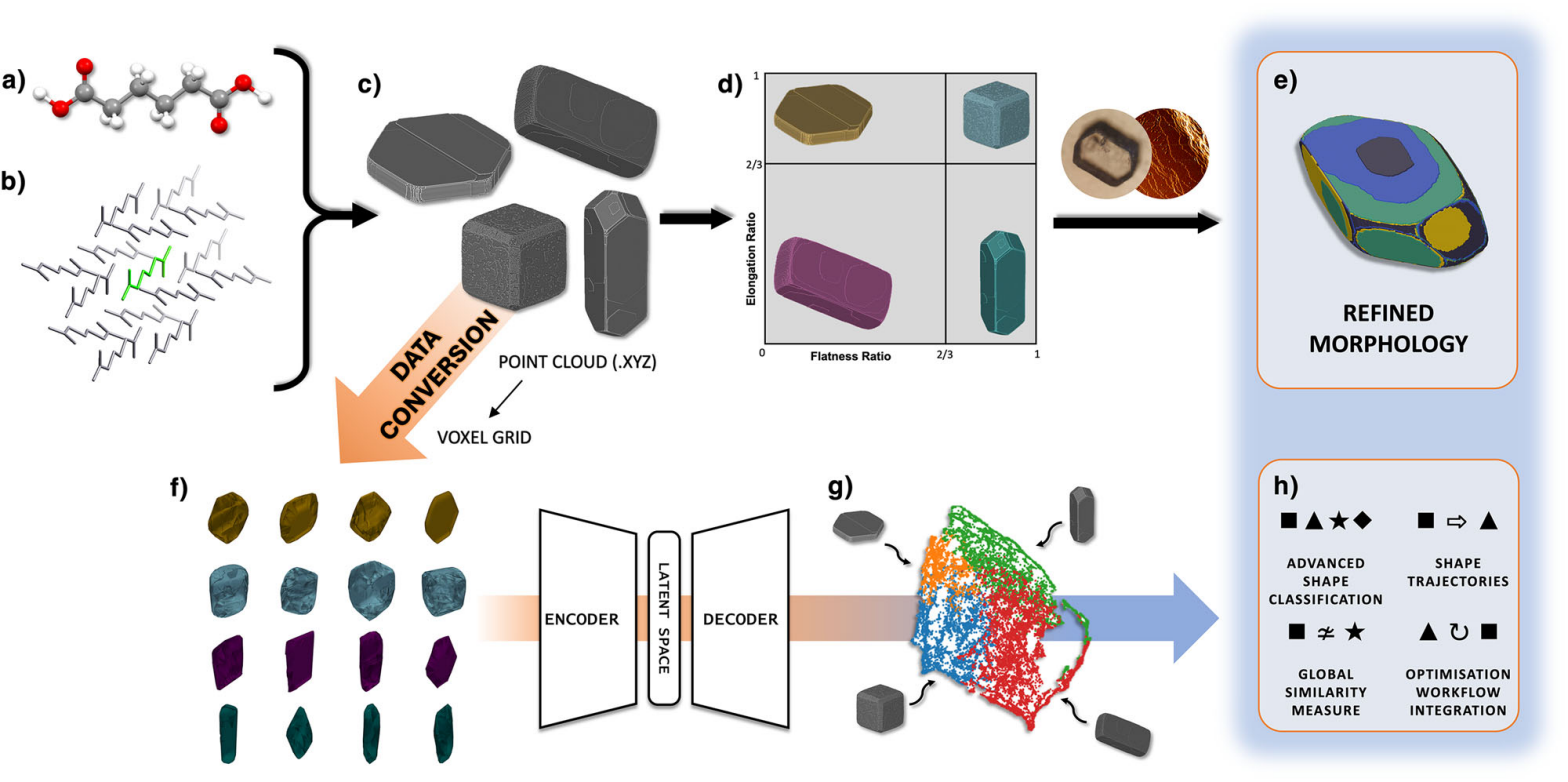
A typical CrystalGrower modelling workflow for investigating a crystal system overlaid with the proposed disentangling autoencoder (DAE) workflow. **a** Input crystallographic information file (CIF) with structural data, (**b**) generation of a net of building units representing key interactions, (**c**) bulk simulations over a parameter space which can be classified using Zingg/aspect ratio analysis (**d**), with validation against experimental data (e.g., optical, electron, or atomic force microscopy) leading to a refined morphology and nanoscale surface topography (**e**). In the proposed DAE workflow, whole particle simulations (**c**) are first downsampled into voxel clouds (**f**), fed through the DAE architecture, and automatically classified (**g**), thus better describing the parameter space, improving on and integrating with existing simulation data analysis capabilities (**h**).

The shape of a crystal can be defined using a range of shape descriptors, ranging from simple metrics such as sphericity^44^ to more complex descriptors such as spherical harmonics^36^. Zingg proposed a method to classify the shape of a particle based on the lengths of orthogonal length, width, and height axes, each of which is classified as either short (S), medium (M), or long (L)^45^. The same principle has been applied to classify crystals by utilising paired or equivalent facets that form the opposing edges of the crystal^46^. By calculating the aspect ratios between these, i.e. S:M and M:L, a Zingg diagram can be created, with crystals falling into one of four categories: blocks, needles, laths, or plates, depending on the value of each aspect ratio. This is shown in Fig. 1d as part of the overall particle simulation workflow using CrystalGrower, which also demonstrates the proposed DAE-based methodology and its integration in the workflow.

The advantages of using the spherical harmonics, *Y*_*lm*_(*θ*, *ϕ*), lie in the completeness and orthonormality of the basis, albeit valid only to shapes represented as a function of spherical coordinates where a one-to-one mapping to the unit sphere is possible (restricted to star domains). Spherical harmonic functions are indexed by the angular quantum number *l* and the magnetic quantum number *m*^47^. These descriptors allow for setting the highest order of *l* to be used for description, herein termed ${l}_{{\rm{max }}}$. It follows that the higher the ${l}_{{\rm{max }}}$, the greater the number of higher-frequency features of the shape are retained within the description.

### Simulated crystal dataset preparation

The dataset used for training and testing the ML model was generated using CrystalGrower, a kinetic Monte Carlo simulation tool designed to model crystal growth with nanoscopic resolution. Here, crystallisation is simulated as a series of reversible transitions between a bulk growth medium and a crystal surface. Starting from a .cif file of the crystal structure, the system is divided into a finite number of surface site types based on their geometrically determined interactions with neighbouring sites, according to the Kossel model of crystal growth^48,49^. The sum of these interactions, adjusted for the energetic cost of solvent removal, defines the free energy of crystallisation (Δ*G*_*s*_) for each site. Supersaturation (Δ*μ*), temperature, and other parameters provide a thermodynamic basis for calculating the probabilities of growth and dissolution events, which are implemented stochastically using a Monte Carlo engine.

During a simulation, growth units replace displaced solvent molecules on the crystal surface, creating distinct site types with varying energies. The output includes a three-dimensional point cloud (.XYZ file) that describes the positions of growth units, capturing the crystal’s full morphology and surface topography at atomic or molecular resolution as well as thermodynamic/site data. Assumptions such as immediate exchanges between crystal and mother phases, unaltered growth unit orientations, and negligible entropy differences between surface sites ensure consistency with thermodynamic principles and allow for a coarse-grained representation of crystal growth. By integrating these elements, this methodology offers a robust platform for studying the interplay between kinetics and thermodynamics in crystal growth, generating data for predictive modelling and advanced analysis.

To build an effective shape classification model, it is important to create a training dataset that exhibits a substantial degree of shape diversity. The expression of different sets of facets, and thus particle shapes, is determined by the simulation parameters, the variation of which leads to their under- or over-expression relative to one another. This means that multiple shapes can occur within a given parameter space. However, not every shape appearing in the parameter space will be observed experimentally, which is why simulations are then validated using experimental data to pinpoint a refined morphology and surface topography.

Owing to the generality of CrystalGrower, in that it can model the growth of any crystal under a range of conditions, this meant selecting chemical systems for which there was a range of known possible morphologies across the thermodynamic parameter space. Thus, adipic acid, benzamide form I, benzamide form III, L-cystine, urea, *γ*-glycine, and paracetamol form I were selected to provide a diverse, somewhat generic representation of crystal shapes. Whilst many of these systems were chosen due to previous experience in their study within the group, urea, L-cystine and *γ*-glycine were specifically chosen to sample molecular crystal structures from outside the commonly observed monoclinic crystal system.

**Fig. 2:**
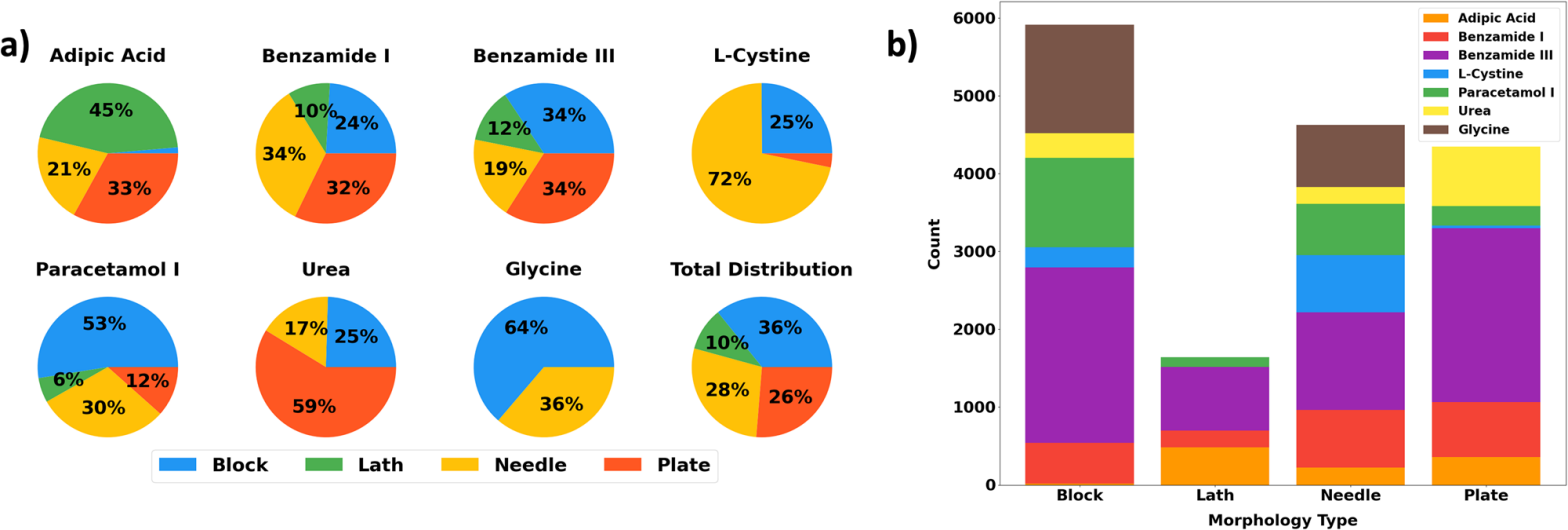
Shape distributions of the training dataset. **a** Grouped by crystal structure, and (**b**) by particle shape as defined by the Zingg classification.

Table 1 details the crystallographic properties of the seven molecular structures that comprise the training dataset. Whilst a majority of the chosen structures are monoclinic, which is reflective of the distribution of space groups in all known molecular crystal structures^50^, they show substantial diversity both in their chemistry and crystallography. As shown in Fig. 2, across the seven molecular systems, 16,522 simulations were carried out, resulting in 5908 blocks, 4346 plates, 4625 needles, and 1643 laths. Variation in the number of simulations per molecule originates from the span of the individual molecular datasets. Tetragonal and hexagonal systems are highly symmetrical, meaning there are fewer energy parameters that can be varied to study the simulation space compared to the less symmetrical monoclinic systems. Smaller increments in the energy parameters used to obtain the molecular crystal datasets result in a denser parameter space with a greater number of points. The simulation of lath-like shapes presented a unique challenge in that these appear to be a much less common shape for molecular crystals to adopt. The inclusion of an alternative crystal packing (known as a polymorph) adopted by benzamide (form III) was aimed at expanding the dataset to add more examples of laths. This was successful, with this structure representing a substantial proportion of the total lath simulations.

**Table 1:** Crystallographic information of molecular structures: structural information for the molecular crystals comprising the training dataset, acquired from ref. ^66^

	Adipic acid	Benzamide-I	Benzamide-III	L-cystine	Urea	*γ*-glycine	Paracetamol-I
Structure							
Formula	C_6_H_10_O_4_	C_7_H_7_NO	C_7_H_7_NO	C_6_H_12_N_2_O_4_S_2_	CH_4_N_2_O	C_2_H_5_NO_2_	C_8_H_9_NO_2_
CSD Refcode	ADIPAC^67^	BZAMID05^68^	BZAMID08^69^	LCYSTI14^70^	UREAXX02^71^	GLYCIN01^72^	HXACAN01^73^
Z	2	4	4	12	2	3	4
Z’	0.5	1	1	0.5	0.25	1	1
Space Group	*P*2_1_/*a*	*P*2_1_/*c*	*P*2_1_/*c*	*P*6_1_22	$P\overline{4}{2}_{1}m$	*P*3_2_	*P*2_1_/*a*
Crystal System	Monoclinic	Monoclinic	Monoclinic	Hexagonal	Tetragonal	Hexagonal	Monoclinic
a, b, c (Å)	10.07, 5.16, 10.03	5.57, 5.04, 21.70	5.06, 5.51, 22.96	5.41, 5.41, 55.96	5.59, 5.59, 4.69	7.04, 7.04, 5.48	12.93, 9.40, 7.10
*α*, *β*, *γ* (∘)	90.0, 137.1, 90.0	90.0, 90.4, 90.0	90.0, 101.3, 90.0	90.0, 90.0, 120.0	90.0, 90.0, 90.0	90.0, 90.0, 120.0	90.0, 115.9, 90.0

To follow the method developed by Zingg to classify the shapes of crystals based on their 3D aspect ratios and provide a manual classification of each structure for validation of the ML model, the dimensions of the simulated crystals were measured. Two methods have been proposed for this: principal component analysis (PCA)^51^ of the point cloud (.XYZ) data of the crystal surface and crystallographic direction analysis (CDA)^52^, which uses the perpendicular distances between parallel crystal surface planes. Both are readily calculated from data within the CrystalGrower output files. It should be noted that the latter presents a unique challenge as a crystal can exhibit multiple sets of surfaces/facets, thus the aspect ratio between any three pairs of facets, in a given area of the parameter space, may not strictly obey the S:M:L criteria as required by Zingg’s method. Thus, for this study, the three largest principal components from PCA were used to calculate the aspect ratios, as they provide a geometrically accurate and standardised aspect ratio calculation. In this work, as all simulated crystal shapes are simple convex polyhedra, an *l*_max_ of was chosen when applying spherical harmonics descriptors to the data.

### DAEs for crystal representation and reconstruction

We use a DAE architecture to learn interpretable representations in a small-dimensional space, referred to as the latent space^53^. In contrast to other autoencoder-based methodologies, the DAE enforces orthogonality in this latent space to achieve disentangled representations, ensuring that each latent variable captures an independent and distinct factor of variation in the data^54^. Whilst a number of linear unsupervised dimensionality reduction techniques, such as PCA, are available, the nonlinear nature of the DAE allows for capturing more complex data representations. By employing both an encoder, which encodes the input data into the latent space, and a decoder, which reconstructs the original inputs, the DAE cannot only learn meaningful representations of the crystals but also reconstruct them from a lower-dimensional representation and visualise the trajectories between two crystals within it. Each of these abilities of the DAE will be reviewed in the following subsections.

### Reconstruction results

**Fig. 3:**
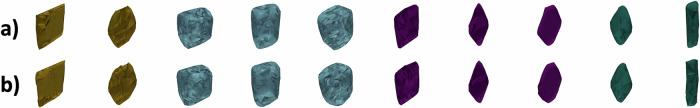
Comparison between input and reconstructed crystals. Reconstruction results demonstrating (**a**) input crystals, and (**b**) reconstructed crystals, with plate morphologies coloured yellow, blocks blue, laths purple, and needles green.

One of the metrics used to ensure that the proposed model learns meaningful features in the latent space is to examine the quality of the reconstructions of the original data when passed through the full autoencoder architecture. High-quality reconstructions confirm that the latent space includes meaningful and relevant information about the crystal shapes, as the decoder arm can generate an accurate reconstruction from the limited information contained within each data point’s latent vector, which is of a substantially smaller dimensionality when compared to the original input. To quantitatively assess the reconstruction quality, we used two metrics: the Structural Similarity Index (SSIM) and the normalised cross-correlation (NCC), both applied to the 3D volumes of the crystal shapes. These metrics range from −1, indicating complete dissimilarity, to 1, indicating perfect similarity. This SSIM quantifies structural similarity by comparing luminance, contrast, and structural information between the original and reconstructed data^55^. Our model achieved an SSIM score of 0.8761. The NCC, which assesses the alignment and similarity between the original and reconstructed data, produced a score of 0.8129. Both scores indicate that the reconstructions preserve a high degree of structural similarity with the original data, suggesting that our model has successfully learned meaningful features in the latent space. Figure 3 shows reconstruction results for crystals of different chemical systems, crystal systems, and shapes appearing in the dataset.

### Analysis of latent dimensions

**Fig. 4:**
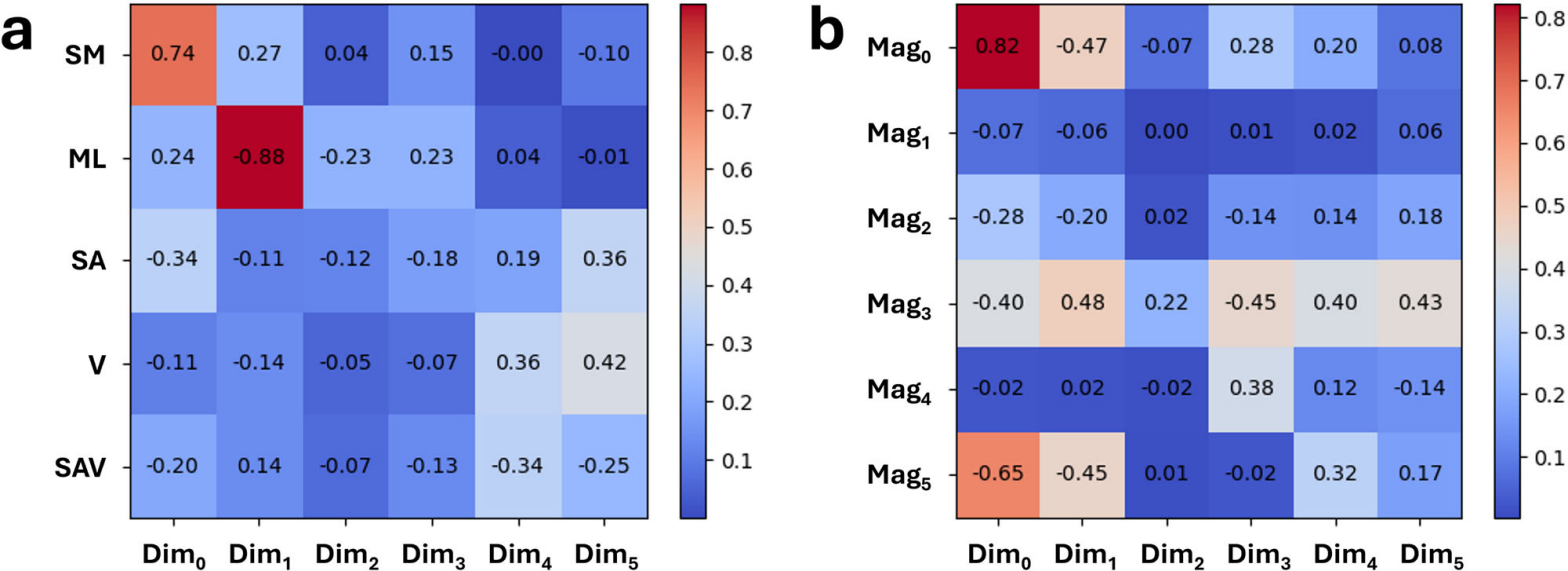
Covariance between latent dimensions and geometric or harmonic shape features. Covariance matrix between the six latent dimensions and (**a**) the ratio between small and medium, the ratio between medium and large, surface area, volume, and the ratio between surface area and volume, and (**b**) the magnitudes of the first six spherical harmonics coefficients.

**Fig. 5:**
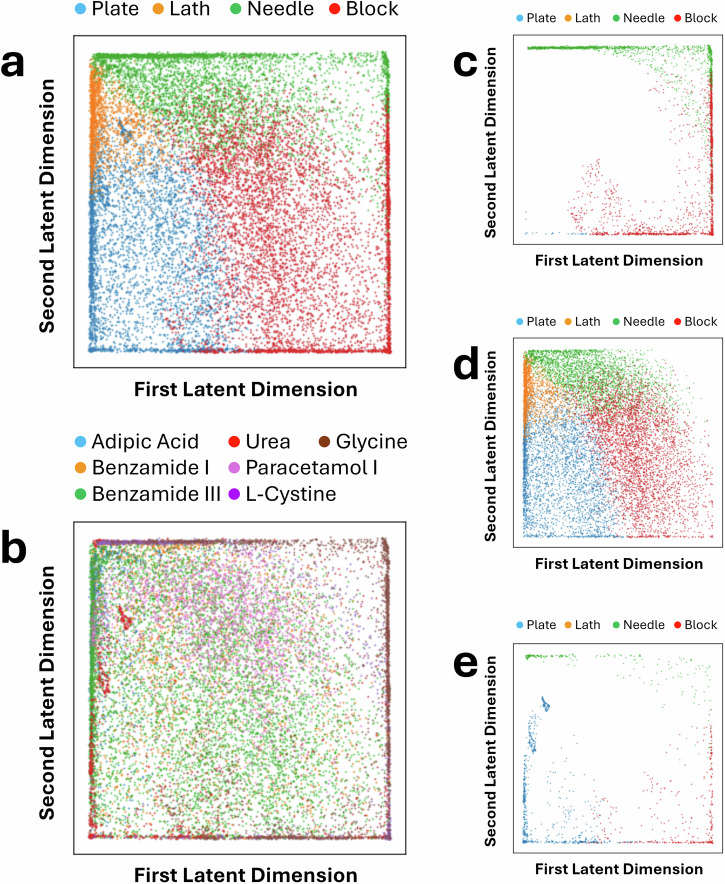
Visualisation of the latent space using the first two dimensions from the DAE. **a** Labelled by shape, and (**b**) labelled by material. Additionally, the latent space is visualised according to crystal systems, namely, (**c**) Hexagonal, (**d**) Monoclinic, and (**e**) Tetragonal.

By analysing the latent space and the latent vectors contained within, we explored the relationship between the latent dimensions and various geometric characteristics of the crystal shapes. An effective ML model should be able to capture geometric relationships between shapes appearing in the dataset, even when the dimensionality of the data is reduced to that of its latent vector. We selected a six-dimensional latent space for this architecture after empirical evaluation. This choice ensures learning sufficient yet minimal features to accurately reconstruct the input data. Specifically, we examined the covariance between the six-dimensional latent variables and quantitative shape descriptors, including the small-to-medium aspect ratio, the medium-to-large aspect ratio, surface area, volume, and the surface-area-to-volume ratio, as shown in Fig. 4a. Our findings indicate that dimension 0 in the latent space shows a strong positive correlation with the small-to-medium aspect ratio of the crystal shapes, which is 0.74. This suggests that this latent dimension effectively captures variations in the relative proportions of the small and medium axes within the crystals. Furthermore, dimension 1 in the latent space shows a high correlation with the medium-to-large ratio, being  −0.88. This implies that dimension 1 predominantly represents variations related to the relative sizes of the medium and large axes in the crystal shapes. Based on these findings, we visualised the latent space using two dimensions that exhibit high correlations with the small-to-medium and medium-to-large aspect ratios, which are geometric characteristics used to describe shape. Figure 5a shows that crystals from the same shape class tend to cluster together, with some overlaps between different shapes, although the boundaries are generally well-defined. Figure 5b presents the same latent space but with points labelled by the chemical system. Since each chemical system can include multiple shapes, the labels inevitably overlap. Notably, urea appears not to be well-clustered, which can be rationalised by the gradual change in morphology from needles to plates that occurs along one axis as thermodynamic parameters are varied.

**Fig. 6:**
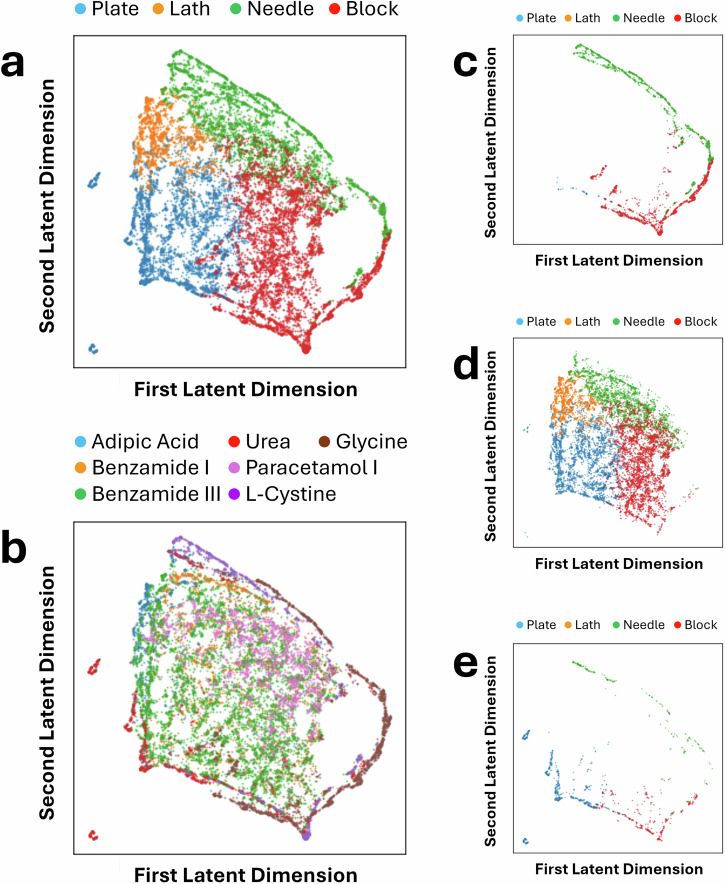
Visualisation of the UMAP projection of the six-dimensional latent space from the DAE. **a** Labelled by shape and (**b**) labelled by material. Additionally, the same UMAP projection is visualised according to crystal systems, namely, (**c**) Hexagonal, (**d**) Monoclinic, and (**e**) Tetragonal.

We also utilised UMAP^56^ to map the six-dimensional latent vectors to two-dimensional vectors for visualisation, as shown in Fig. 6. Since UMAP uses all six dimensions, the resulting visualisation provides clearer boundaries between shapes when compared to using only two dimensions. Again, urea appears as an outlier, appearing primarily at the edge of the mapping, or as individual clusters separated from the main body of the latent space.

Furthermore, we conducted the same experiments using a *β*-VAE^57^ and visualised its latent space in the same manner as the DAE. These mappings are presented in Supplementary Note 1. When considering the two dimensions that exhibit high correlations with the small-to-medium and medium-to-large aspect ratios, as shown in Supplementary Fig. S1, the results appear similar to those of the DAE. However, when examining the UMAP projection across all latent dimensions, as shown in Supplementary Fig. S2, the DAE exhibits clearer clustering between different shapes, suggesting that its orthogonal constraints help better distinguish morphological variations. Once again, urea does not appear well-clustered in the *β*-VAE.

### Comparison with spherical harmonics descriptors

From Fig. 4b, we are able to derive important relationships between the spherical harmonic coefficient space, the autoencoder latent space, and their relationship to physical shape metrics observed in Fig. 4a. Since each spherical harmonic coefficient can be described by its two quantum numbers *l* and *m*, each coefficient is indexed as ${Y}_{l}^{m}$. The first six spherical harmonic coefficients (real, *m* ≥ 0) referenced in Fig. 4b (*Mag*_0→5_) thus represent ${Y}_{0}^{0}$, ${Y}_{1}^{0}$, ${Y}_{1}^{1}$, ${Y}_{2}^{0}$, ${Y}_{2}^{1}$ and ${Y}_{2}^{2}$. *Mag*_0_, being the unit sphere, therefore represents the average particle radius. *Mag*_1_ and *Mag*_2_ represent dipole-like shape asymmetries; for example, *Mag*_1_ informs on the span of the shape along the Cartesian Z axis, whereas *Mag*_2_ informs on the particle’s span along the Y axis. *Mag*_3→5_ represents quadrupolar shape features such as elongation or flattening in specific directions. Specifically, *Mag*_3_ represents elongation along Z with contraction in the XY plane, while *Mag*_4_ and *Mag*_5_ relate to the particle’s size in the YZ diagonals and along the XY directions, respectively. These relationships are also evident in the reconstructions presented in Supplementary Note 1.

*Mag*_0_ has the strongest correlation to any of the latent dimensions, being both positively correlated with Dim_0_ and negatively correlated with *Dim*_1_, but it only forms weak correlations with other latent dimensions. A strong correlation is also seen in *Mag*_5_, where it exhibits a negative correlation with several latent dimensions, indicating that the DAE latent space encodes shape features in a way that is not aligned with the spherical harmonics. Analysis shows that *Dim*_0_ and *Dim*_1_ exhibit the strongest correlations with the spherical harmonic coefficients. In particular, *Dim*_0_ and *Dim*_1_ show strong correlations with *Mag*_0_, *Mag*_3_, and *Mag*_5_, suggesting these first two latent variables capture primarily aspect ratio and overall size distortions. This observation aligns with the results shown in Fig. 4a, supporting the conclusion that these dimensions encode the most information about aspect ratio. The latent dimensions (*Dim*_3→6_) show weak or mixed correlations with different spherical harmonic terms, implying that they may encode more complex, nonlinear shape features. Again, this is also reflected in Fig. 4a, where no strong correlation between the latter dimensions is seen with respect to the chosen physical shape metrics. Overall, the presence of strong correlations between specific spherical harmonics and DAE dimensions suggests that certain latent variables effectively capture spherical harmonic features. However, more controlled studies would be required to accurately probe the overlap between these spaces.

**Fig. 7:**
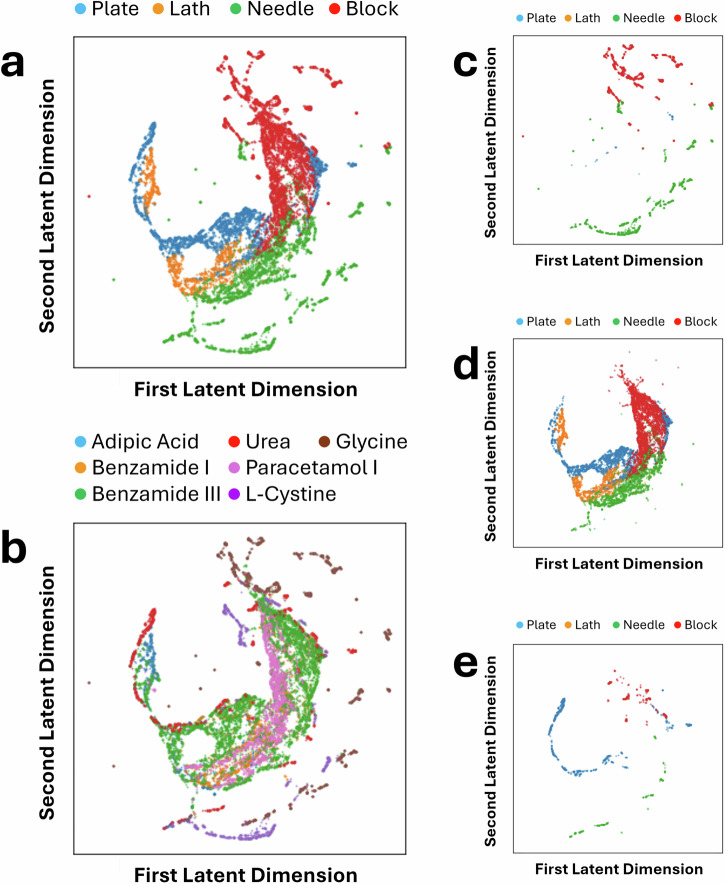
Visualisation of the UMAP projection of the spherical harmonic coefficients. **a** Labelled by shape, and (**b**) labelled by material. Additionally, the same UMAP projection is visualised according to crystal systems, namely, (**c**) Hexagonal, (**d**) Monoclinic, and (**e**) Tetragonal.

Figure 7 provides an additional visualisation of the (66-dimensional) spherical harmonic coefficient space, reduced to two dimensions using UMAP. Compared to the *β*-VAE and DAE UMAP latent spaces, in the spherical harmonic UMAP, the separation of points based on crystal system or chemical system is less clear, and features blurred boundaries. Whilst the DAE shows grouping of points according to shape class in the form of broader regions rather than sharply defined clusters, the spherical harmonic UMAP also forms comparatively well-defined regions compared to that of the *β*-VAE latent space.

Interestingly, the *β*-VAE space forms distinct clusters based on chemical systems, whilst both the spherical harmonic space and the DAE UMAP space seemingly retain less information on structure-shape relationships. To further rank these models would require additional methodological considerations beyond the scope of this study. Another minor observation is that block-shaped crystals appear to form a tighter group, which may be related to the ability of SHSDs to describe shapes that are morphologically closer to a sphere more accurately. Overall, these visual assessments allow for a qualitative understanding of the different shape descriptor models. For example, it becomes clear that both the DAE and *β*-VAE representations are able to encode important shape features in fewer dimensions compared to a physics-based descriptor such as spherical harmonics.

### Morphological dependency on crystal system

Given that crystal structures have a limited set of allowed shapes based on their underlying symmetry, the output of the DAE can be further investigated and verified through comparison to results expected from crystallographic theory. The crystal systems for all crystal structures studied are summarised in Table 1, with a total of three different systems across the dataset: hexagonal, monoclinic, and tetragonal. Figure 5b–e demonstrates how filtering the data from the first two dimensions of the latent space can provide additional insight into crystal morphologies.

Figure 5c–e demonstrates that the model does follow the expected outcomes from crystallography. Hexagonal and tetragonal crystal systems both show no lath morphologies, which is expected due to the two axes of the crystal system being of equal length, which only allows for asymmetric variation along one axis for observed morphologies. In monoclinic systems, however, all axes are of unequal length, meaning that each axis can vary asymmetrically when a crystal is grown, reflected by the appearance of lath morphologies in the latent space mappings.

Of note is the near absence of plate morphologies in the hexagonal systems (Fig. 5c, L-cystine and *γ*-glycine), which intuitively should appear as a block flattened along the variant axis, compared to a needle, which is simply an extension along the same axis. This absence can be attributed to the angle (*γ*) between the equivalent a and b axes in the crystal system, which is 120° rather than the orthogonal 90° seen in tetragonal systems (Fig. 5e, urea). As PCA will use a set of orthogonal Cartesian axes derived from the lengths of the particle point cloud, these can vary from the unit cell axes in fractional space. For a cubic particle, the short, medium, and long PCA axes would correspond to the edge of a cube (a), the diagonal across a cube face ($\sqrt{2}* a$), and the body-diagonal across the cube ($\sqrt{3}* a$), respectively. A cubic particle would therefore be classed as a block in the Zingg system, due to the S:M and M:L aspect ratios both being greater than 0.66. However, for a hexagonal system, the square particle faces become rhombuses, meaning there are multiple face-diagonal distances caused by the acute and obtuse angles of the rhombus ($a\sqrt{2+2cos\alpha }$ and $a\sqrt{2-2cos\alpha }$ where *α* = 60°). The body-diagonal becomes longer, and the shorter of the face-diagonal lengths shrinks relative to the edge length. Therefore, the “long" length as defined by Zingg is likely to be extended relative to the other lengths, causing more crystals to be classified as needles rather than plates or blocks. Needles and blocks are well represented in the tetragonal system, supporting the conclusion that this effect is driven by the angle in the unit cell, rather than the unequal axis lengths.

In contrast to the monoclinic systems (Fig. 5d), which comprise the bulk of the data, the hexagonal and tetragonal system morphologies tend to cluster at the edges of the plot. Again, this is a reflection of the number of degrees of freedom available to the materials within these crystal systems, with smoother transitions between morphologies. Urea is the main outlier, with morphologies that push into the lath region of the plot (Fig. 5b). This was attributed to the rapid transition from plates to needles in this system within the dataset when changing the free energy of crystallisation (Δ*G*_*s*_) parameters. The UMAP projection in Fig. 6e also shows the tendency for urea morphologies to separate from the bulk of the data, which may be evidence of poor clustering. However, the separation of the hexagonal and tetragonal morphologies from monoclinic already provides additional information when compared to aspect ratio analysis alone.

It was hypothesised that differences could be observed between the two forms of benzamide, as form I could be considered a quasi-orthorhombic crystal system, in that the angles of its unit cell are all close to 90°, demonstrating that there are even borderline classification cases in fundamental crystallographic theory. Despite this, both polymorphs distribute similarly throughout the latent space, as all cell axes are of unequal length, meaning both possess the same number of degrees of freedom for their respective monoclinic and orthorhombic forms. However, for more crystallographically distinct polymorphs, greater separation would be expected.

### Shape evolution trajectories

**Fig. 8:**
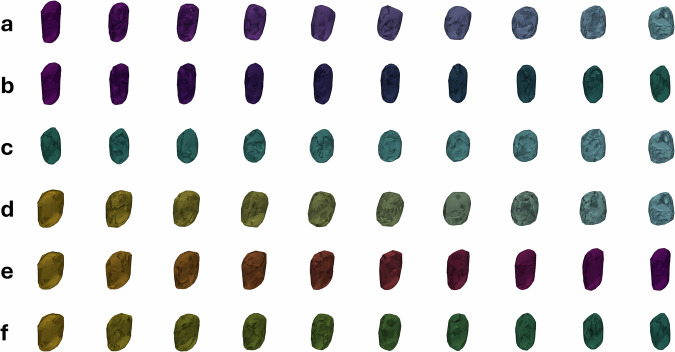
Interpolated trajectories between crystal morphologies in the latent space. Trajectories depicting shape evolutions using the decoded latent vectors appearing in the latent space between the centres of (**a**) lath and block classes, (**b**) lath and needle classes, (**c**) needle and block classes, (**d**) plate and block classes, (**e**) plate and lath classes, and (**f**) plate and needle classes, with plate morphologies coloured yellow, blocks blue, laths purple, and needles green.

**Fig. 9:**
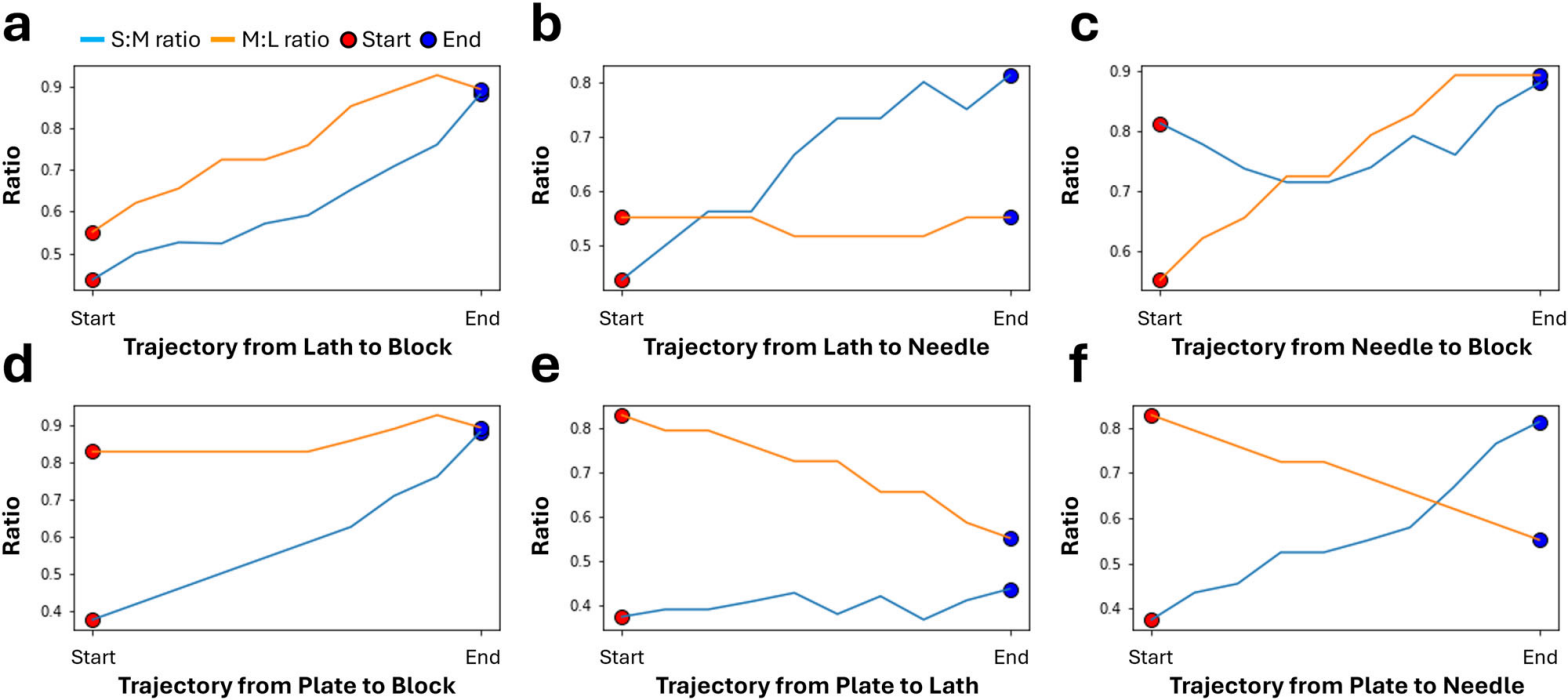
Changes in particle aspect ratios across shape trajectories between crystal class centres. Quantitative depiction of S:M and M:L aspect ratio evolution across shape class trajectories between (**a**) lath and block classes, (**b**) lath and needle classes, (**c**) needle and block classes, (**d**) plate and block classes, (**e**) plate and lath classes, and (**f**) plate and needle classes.

The use of an autoencoder-based methodology allows for the ability to not only capture the complex relationships between different crystal shapes in the latent space, but also to visualise the trajectories of transformations from one shape or class to another. By identifying the centre of each shape class (plate, lath, block, and needle) in the latent space, we can use the decoder to reconstruct the trajectories that represent the smooth transition between these shapes. In Fig. 8, we present the trajectories of changes from one shape class to another within the latent space. These trajectories provide insight into the continuous morphing process between different crystal shapes, which would be difficult to observe or quantify directly in the original feature space. Point cloud data alone, even when analysed by aspect ratio, cannot capture the broader geometric and crystallographic patterns that emerge across the entire dataset. The DAE approach overcomes this limitation by uncovering the fundamental relationships between different data points and, by extension, crystal morphologies, thus providing a global measure of similarity across the dataset. To further quantify these shape transformations, we introduce Figure 9, which depicts the evolution of aspect ratio along these shape class trajectories. Each subfigure in Fig. 9 directly corresponds to its counterpart in Fig. 8. By incorporating quantitative measures, Fig. 9 complements the visualised trajectories in Fig. 8, offering deeper insights into the structural changes that occur as crystals transition between different shape classes. The quantitative changes in S:M and M:L aspect ratios across trajectories correspond to those expected according to Zingg analysis, and thus provides the magnitude of change across individual trajectories.

By visualising these shape transformation trajectories, our model proves applicable not only to dimensionality reduction but also in revealing how crystal morphologies systematically evolve across a given parameter space. Such transitions between shapes or their classes could provide insights for mechanism studies when analysing bulk simulations of a single-crystal system. This is because a given trajectory would follow changes in one or more simulation parameters, which are physically interpretable according to the CrystalGrower model.

## Discussion

We opted for a DAE over the *β*-VAE^58^ because the DAE is specifically designed for disentanglement by enforcing orthogonality in the latent space. This orthogonality constraint ensures that the latent dimensions remain independent, allowing for a clearer separation of features. By contrast, due to their probabilistic nature, *β*-VAEs can sometimes produce overlapping latent variables, which leads to less distinct feature separation, particularly when feature variances differ. We have shown that two active features in the latent space are highly correlated with the aspect ratios of the crystals. The third feature did not demonstrate a high correlation with other physical quantities, such as the surface area or volume of the crystals. It is possible that this feature could represent a combination of multiple physical or geometric properties, which is why it was not possible to find a direct correlation with any one of the physical features provided in the dataset. In this work, the DAE architecture was used to learn features for crystal shape analysis. However, because the layers in the encoder and decoder were not explicitly designed to be invariant to 3D voxel data, it was necessary to align the objects prior to training. For future work, developing a model that incorporates equivariance and invariance to 3D rotations would allow for feature learning directly from unaligned data. One possible route to achieving this could be the use of group equivariant convolutional neural networks, which would leverage the symmetries inherent in the data^59,60^. This could improve generalisation and performance, particularly in tasks involving arbitrary rotations of 3D shapes.

Additionally, in varying the various free energies of crystallisation, Δ*G*_*s*_, that comprise the major thermodynamic simulation parameters, a range of different shapes can be obtained. A drawback of Zingg’s methodology is the idea of a hard numerical border between shape classifications when the range of shapes appearing within a given parameter space can be considered continuous. It would be expected that variation along a particular parameter’s ’trajectory’ causes a transition between shape types, e.g., from block to needle when such a parameter change causes elongation along one axis only. As shown in the shape distribution pie charts provided in Fig. 2, an example of this would be glycine, where only block- or needle-like morphologies are observed. Such an effect could explain the predominant appearance at the edge of the latent space mappings. Conversely, in the case of urea, a majority plate system, a more limited range of accessible shapes within the parameter space may explain the sparsity seen in the tetragonal latent space mappings, although further investigation would be needed to confirm this.

It is also possible that a number of different shapes, whilst distinct in terms of faceting or overall geometry, may fall under a similar numerical Zingg classification. This could be thought of in terms of the difference between a cube and a rhombohedron, both of which would be classified as blocks with broadly similar S:M and M:L aspect ratios  >0.66, yet different faceting and thus potentially dissimilar crystallochemical properties. By using Zingg analysis alone, a crystallographic or chemical distinction between these shapes cannot necessarily be made. However, by using the DAE methodology, one would reasonably expect these two shapes to appear distinct and separated in the latent space, thus giving a more useful overall description of particle shape relative to the parameter space surveyed.

Furthermore, whilst it can be argued that the Zingg notation of block, needle, plate, and lath can describe the vast majority of shapes to an arbitrary level of detail, their non-specificity means that the diversity and nuance in morphologies comprising each category are not captured, as demonstrated in this work. Future work could explore whether the DAE architecture employed here would be able to classify the shapes comprising the training dataset to the same level of accuracy, should a greater range of shape descriptors of a higher specificity be utilised.

Another point to note is the lack of standardisation of crystal morphology descriptors. In developing our dataset classification methodology, we were required to rely on an appropriate albeit archaic classification, namely particle aspect ratio (Zingg) analysis, which was first proposed in 1935. Although there are clear, mathematically derived conventions within crystallography on molecular symmetry (space groups and point groups), which directly influence the allowed morphologies of a given structure, there is currently no equivalent universal standard for crystal shapes. Black and Seton have recently highlighted the shortage of high-quality morphological data for organic crystals, proposing a method to systematically link historical data from P. von Groth’s Chemische Kristallographie with modern crystal structures in the Cambridge Structural Database (CSD) by matching unit cells and storing the data in a standardised “morphology.cif” format ^61^. Additionally, in data mining a major structural database, Wilkinson et al. noted variations in the user-defined morphological labels applied to entries ^13^. It remains to be seen whether consensus can be reached on how to best describe crystal shapes in a manner compatible with existing databases and practices within the field.

In the age of data-driven science, many different statistical or dimensionality reduction techniques can be used in the analysis of large and complex datasets. By utilising a kinetic Monte Carlo model with a finite number of (directional) free energy parameters, tuning particle morphology by adjusting experimental conditions to favour or disfavour chemical interactions along these directions can result in greater control over particle design and maximise relevant chemical performance. Similarly, control over relative growth rates along directions corresponding to different aspect ratios can make a range of previously inaccessible morphologies producible. The DAE demonstrated here is a substantial improvement on existing simulation analysis workflows, which currently produce individual Zingg diagrams based on each free energy of crystallisation (Δ*G*_*s*_) parameter and do not provide a wider, global context of how data points are clustered.

When the DAE is employed, significantly more complex relationships lying within the dataset, whether geometric or crystallographic, can be elucidated, and a relative measure of similarity/dissimilarity between shapes appearing in the parameter space is provided via latent space mappings. The results presented in this paper prove that additional, relevant insight into crystal growth processes can be gained via the use of ML and SHSDs.

## Methods

### CrystalGrower simulations

Each simulated crystal in the dataset was run for 3,000,000 iterations (where each iteration corresponds to a single growth or dissolution event) at 25 °C. Energy parameters for interactions within the crystal structures were varied to produce crystals adopting a diverse set of morphologies for study. A high value (100 kcal mol^−1^) of the thermodynamic driving force, Δ*μ*, was set for over two-thirds of the simulation run to ensure the growth of a sufficiently large crystal for study, before equilibrating over the course of a 100,000 iteration period and remaining at equilibrium for the final 400,000 iterations to reach an equilibrium shape. Data were output in .XYZ point cloud format, which was then normalised to give a coordinate set in the range of 0–1.

Crystal growth simulations of glycine and paracetamol form I were carried out using CrystalGrower 1.4.0 (Linux) on the CSF3 (Computational Shared Facility 3) at The University of Manchester, composed of Intel Haswell, Broadwell, or Skylake cores. Crystal growth simulations of all other systems were carried out using CrystalGrower 1.4.0 (Linux) on the CSF4 (Computational Shared Facility 4) at The University of Manchester, composed of Intel Cascade Lake CPUs.

Individual CrystalGrower calculations were performed in serial, with simulation batches spread over CPU cores by the job scheduling systems (Sun Grid Engine - SGE on CSF3, and SLURM on CSF4).

### Dataset conversion for model input

Using Python, the .XYZ output of the simulated crystal surface was read into a 2D array. The centre of the point cloud was moved to the origin of the coordinate system, and the centred set of vertices was normalised by dividing each vertex by the maximum Euclidean norm of all centred vertices. The Python package scikit-learn^62^ was used to execute PCA on the normalised vertices, and the ratio of the singular values of each of the components was used as the S:M:L aspect ratio. This alignment is necessary because our model is based on 3D-convolutional layers, which are not rotationally equivariant or invariant. A shape file was produced for each simulation containing the crystal’s shape label (block, lath, needle, or plate) and the numerical S:M and M:L aspect ratios. The QHull algorithm^63^ was used to calculate a convex hull of the simulated crystal shape, from which the surface area and volume were calculated. These parameters, plus a calculated surface area/volume (SA/Vol) ratio, were included in the shape file as other potentially useful features for shape description.

The raw point clouds usually lack neighbourhood structure, making it difficult to extract semantic, geometric, or topological information from images. Voxels, by contrast, are abstracted 3D units with pre-defined volumes, positions, and attributes, which naturally encode the spatial distribution of 3D shapes. Therefore, the .XYZ data was converted to voxel format. Pad zero values were added to the edge of each voxel cloud to ensure all voxels were of the same size. Voxel clouds were then downsampled to a size of 32 × 32 × 32 by applying a max function to non-overlapping blocks of the voxel. This was done using the block_reduce function in the scikit-image library in Python.

### Model construction

A DAE architecture with 3D-convolutional layers is used to learn disentangled representations of the range of crystal shapes. The DAE architecture enforces orthogonality constraints on the latent variables through Euler encoding transformation, ensuring linear independence between dimensions in the representation space^53^. By reconstructing the original inputs through the decoder network, the method ensures that the latent variables represent the input data in a lower-dimensional latent space with minimal correlation between features.

The model architecture and training regime are outlined in Supplementary Notes 3 and 4, respectively. The model was trained using a single NVIDIA Tesla V100 GPU from the PEARL system at the Science and Technology Facilities Council (STFC), which consists of two NVIDIA DGX-2 nodes. The code was implemented with PyTorch, which enabled efficient GPU acceleration.

### Spherical harmonics descriptors

Throughout this study, the method introduced by Spackman in describing Hirshfeld surfaces has been utilised to describe the simulated 3D crystal shapes^7,64,65^. CrystalGrower simulations in point cloud form were processed in Python to obtain the spherical harmonic coefficients for each simulation. The convex hull of each simulated crystal shape, represented as a 2D coordinate array, was computed using the QuickHull algorithm implemented in the SciPy package^63^. Using the equations of the hull, the chmpy Python package was then used to calculate the spherical harmonic coefficients representing the shape. The number of spherical coordinates sampled and the number of calculated coefficients directly correlate to the *l*_max_ parameter.

Spherical harmonic decomposition of the crystal shapes was conducted with a truncation parameter *l*_max_ = 10, consistent with established protocols for shape-matching applications in the literature. This is presented visually in Supplementary Note 1. It is important to note that the spherical harmonic coefficients obtained are described in a consistent Cartesian coordinate system. Any comparisons made between the coefficients were direct comparisons of the real spherical harmonic coefficients without any rotational invariance.

## Supplementary information

Supplementary Information

## Data Availability

Datasets (in voxel format used for model training) relating to this work are available on request from the corresponding authors or CrystalGrower Ltd. (team@crystalgrower.org).

## Code availability

Code relating to this work is available at https://github.com/stfc-sciml/crystal_dae.
