# Supplementary material for: Disentangling autoencoders and spherical harmonics for efficient shape classification in crystal growth simulations
Source: Commun Phys. 2025 Jul 2;8(1):272. doi: 10.1038/s42005-025-02129-7 (PMC12221979; doi:10.1038/s42005-025-02129-7)
Supplement: Supplementary file 1 — Supplementary Information [file 42005_2025_2129_MOESM1_ESM.pdf]

Disentangling Autoencoders and Spherical Harmonics for Efficient Shape Classification in Crystal Growth Simulations—*Supplementary Notes*

Jaehoon Cha[1], Steven Tendyra[2,3], Alvin J. Walisinghe[2,4], Adam R. Hill[2,3], Susmita Basak[1], Peter R. Spackman[2,4], Michael W. Anderson[2,3], Jeyan Thiyagalingam[1]

[1] Scientific Computing, Rutherford Appleton Laboratory, Science and Technology Facilities Council, Harwell Science and Innovation Campus, Didcot, OX11 0QX, United Kingdom.

[2] CrystalGrower Ltd., Core Technical Facility, 46 Grafton Street, Manchester, M13 9NT, United Kingdom.

[3] Department of Chemistry, The University of Manchester, Oxford Rd, Manchester M13 9PL, United Kingdom.

[4] School of Molecular and Life Sciences, Curtin University, PO Box U1987, Perth, WA 6845, Australia.

S1. Supplementary Note 1: Comparison of DAE with β-VAE

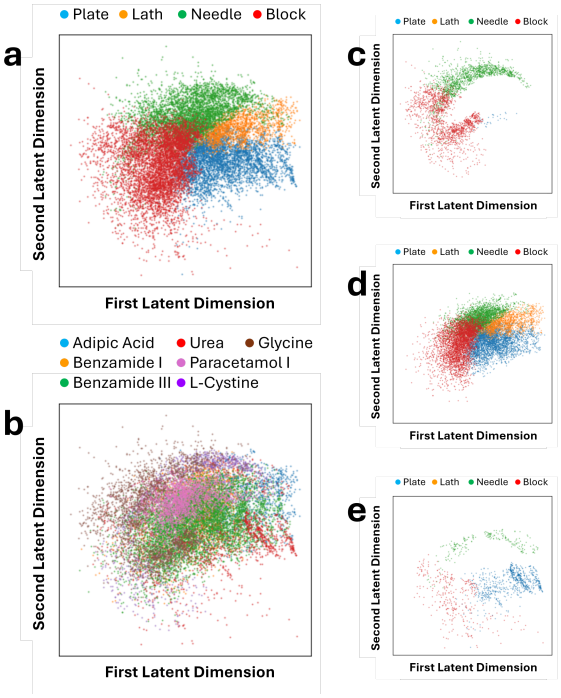

Figure S1: Visualisation of the latent space using the first two dimensions from the variational autoencoder (β-VAE): (a) labelled by shape and (b) labelled by material. Additionally, the latent space is visualised according to crystal systems, namely, (c) Hexagonal, (d) Monoclinic, and (e) Tetragonal.

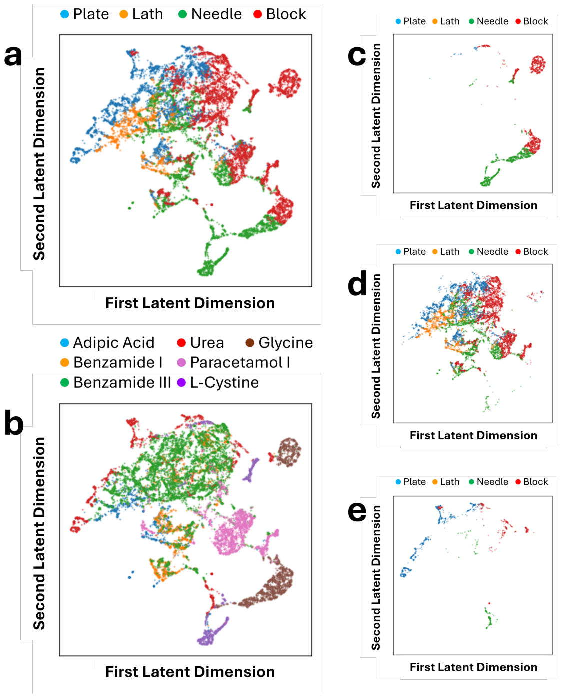

Figure S2: Visualisation of the UMAP projection of the six-dimensional latent space from the variational autoencoder (β-VAE): (a) labelled by shape and (b) labelled by material. Additionally, the same UMAP projection is visualised according to crystal systems, namely, (c) Hexagonal, (d) Monoclinic, and (e) Tetragonal.

S2. Supplementary Note 2: Spherical harmonics shape descriptors (SHSDs)

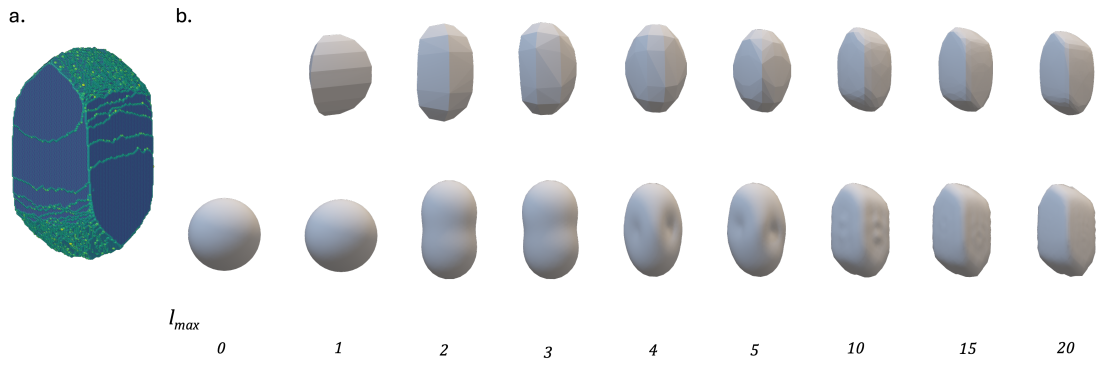

Figure S3: Reconstruction of a reference crystal shape with two different surface reconstruction approaches based on spherical harmonic coefficients: (a) The convex hull approach illustrates reconstructions where the surface is defined by computing the convex hull of the reconstructed point cloud. (b) The icosphere-based method presents reconstructions where an icosphere mesh is deformed according to the evaluated spherical harmonic expansion. Both methods are shown across increasing l_{max} values, demonstrating the convergence of the reconstructed shape towards the reference morphology.

The maximum spherical harmonic order parameter, l_{max} determines the number of coefficients used to describe a particular shape. In the literature within the chemical sciences, it is common to see lower l_{max} values (<10), as this in most cases has been sufficient in describing the necessary features of the shapes in question. However, it is clear that in other fields ranging from computer graphics to astronomy, l_{max} values are used that are in the thousands to millions of coefficients [1, 2].

In this study, we have selected an l_{max} of 10 to represent the simulated crystal shapes. This was deemed suitable, both through what has been reported in the literature and expertise in using the CrystalGrower workflows, see Supplementary Figure S3.

In Figure S3, it can be seen that a delicate balance is preserved between the computational cost (albeit low) and sufficient resolution in the description when l_{max} is set to 10. The use of higher l_{max} values contributes to better-defined edges and corners. However, the increasing number of additional coefficients, combined with diminishing gains in resolution, may introduce more noise, particularly during dimensionality reduction techniques such as UMAP.

S3. Supplementary Note 3: DAE vs SHSDs

Table S1: Comparison of DAE models and spherical harmonics for shape description.

**Feature**	**DAE Model**	**Spherical Harmonics Descriptors**	**Complementarity in Applications**
**Representation**	Learns latent features automatically from data	Uses predefined mathematical basis functions	DAEs capture complex, data-driven patterns; spherical harmonics provide interpretable, structured representations.
**Interpretability**	Latent features may not have direct physical meaning but can be investigated for coupling with physical features	Coefficients related to lower l values ($l < 2$) directly encode specific shape features; *e.g.* $l = 0$: average radius, $l = 1$: average ellipsoid	Both methods can express physical features.
**Dimensionality Reduction**	Efficient in compressing high-dimensional data	Limited: depends on the order of l used	DAE learns minimal but sufficient information to reconstruct original data; spherical harmonics offers controlled, physics-based reduction.
**Robustness to Noise**	Can learn robust representations from noisy data	Sensitive to noise, especially with high-order terms	DAEs filter out high-frequency noise that doesn't contribute to meaningful patterns.
**Computational Cost**	High: requires training on large datasets	Moderate: depends on number of coefficients used	DAEs require initial training but generalize well; spherical harmonics are efficient for specific problems like direct 3D shape comparison.
**Application**	General-purpose, data-driven shape learning	Well-suited for analytically defining crystal morphologies	Combining both leverages data-driven insights while maintaining physical interpretability.

S4. Supplementary Note 4: Model architecture

Table S2: Architecture of the DAE's encoder and decoder arms.

Encoder ↓	Decoder ↑
Input 1×32×32×32 image	3×3×3 1 Conv ↓, Sigmoid
4×4×4 16 Conv ↓, BN, LReLU	4×4×4 1 Conv ↑, BN, LReLU
4×4×4 32 Conv ↓, BN, LReLU	4×4×4 16 Conv ↑, BN, LReLU
4×4×4 64 Conv ↓, BN, LReLU	4×4×4 32 Conv ↑, BN, LReLU
4×4×4 128 Conv ↓, BN, LReLU	4×4×4 64 Conv ↑, BN, LReLU
FC 128	FC 1024, LReLU
FC 6	FC 128, LReLU

The model architecture is summarised in Table S2. The encoder takes a voxel cloud of size 32×32×32 as the input. There are 4 3D-convolutional layers with 16, 32, 64, and 128 channels, respectively. The convolutional filter size is $4 \times 4 \times 4$ for each layer and each convolutional layer is followed by a batch normalisation (BN) layer. The Leaky Rectified Linear Unit (LReLU) activation function is used for each of these layers. We use a stride of 2 in each convolutional layer to downsample the voxel cloud. There are two fully connected (FC) layers following the last convolutional layer. The first FC layer had 128 nodes and the second FC layer has nodes equal to the number of features in the latent space. The output of the second FC layer is then transformed and passed through a normalisation layer, an interpolation layer, and an Euler layer before being fed into the first layer of the decoder. Subsequently, the output of the Euler layer is fed into two FC layers, comprising 128 and 1024 nodes, respectively, to reconstruct the input data.

Symmetrical to the encoder, the output of the FC layer is reshaped and passed into a 3D transposed convolutional layer. In the decoder, there are 4 3D-transposed convolutional layers with 64, 32, 16 and 1 channels with strides of 2, respectively. Each of those layers is followed by a BN layer and activated with the LReLU function. Following this, there is a convolutional layer with a $3 \times 3 \times 3$ filter with a Sigmoid activation function.

S5. Supplementary Note 5: Training configuration

We used the Adam optimiser with a learning rate of 0.0001 and a batch size of 64 for training all models. All models were trained for 300 epochs with Binary Cross-Entropy (BCE) as the loss function. Both DAE and β-VAE incorporate a hyperparameter to encourage disentanglement, as introduced in their respective formulations: α for DAE and β for β-VAE. In our experiments, we set $\alpha = 0.05$ and $\beta = 16$.

Supplementary References

[1] Will J. Percival, Daniel Burkey, Alan Heavens, Andy Taylor, Shaun Cole, John A. Peacock, Carlton M. Baugh, Joss Bland-Hawthorn, Terry Bridges, Russell Cannon, Matthew Colless, Chris Collins, Warrick Couch, Gavin Dalton, Roberto De Propris, Simon P. Driver, George Efstathiou, Richard S. Ellis, Carlos S. Frenk, Karl Glazebrook, Carole Jackson, Ofer Lahav, Ian Lewis, Stuart Lumsden, Steve Maddox, Peder Norberg, Bruce A. Peterson, Will Sutherland, and Keith Taylor. The 2df galaxy redshift survey: spherical harmonics analysis of fluctuations in the final catalogue. *Monthly Notices of the Royal Astronomical Society*, 353(4):1201–1218, October 2004. ISSN 1365-2966. doi: 10.1111/j.1365-2966.2004.08146.x. URL `http://dx.doi.org/10.1111/j.1365-2966.2004.08146.x`.

[2] Moritz Rexer and Christian Hirt. Ultra-high-degree surface spherical harmonic analysis using the gauss–legendre and the driscoll/healy quadrature theorem and application to planetary topography models of earth, mars and moon. *Surveys in Geophysics*, 36(6):803–830, October 2015. ISSN 1573-0956. doi: 10.1007/s10712-015-9345-z. URL `http://dx.doi.org/10.1007/s10712-015-9345-z`.
